# Supplementary material for: Multi‐dataset identification of innovative feature genes and molecular mechanisms in keratoconus
Source: J Cell Mol Med. 2024 Sep 19;28(18):e70079. doi: 10.1111/jcmm.70079 (PMC11412914; doi:10.1111/jcmm.70079)
Supplement: Supplementary file 2 — Material S2. [file JCMM-28-e70079-s002.zip › 7.GSEA analysis/4.MYRF/KEGG_INOSITOL_PHOSPHATE_METABOLISM.html]

Details for gene set KEGG\_INOSITOL\_PHOSPHATE\_METABOLISM[GSEA]

|  || Dataset | MYRF.group.cls#h\_versus\_l |
| Phenotype | group.cls#h\_versus\_l |
| Upregulated in class | l |
| GeneSet | KEGG\_INOSITOL\_PHOSPHATE\_METABOLISM |
| Enrichment Score (ES) | -0.37974232 |
| Normalized Enrichment Score (NES) | -1.4973248 |
| Nominal p-value | 0.07157464 |
| FDR q-value | 0.21163149 |
| FWER p-Value | 0.766 |
Table: GSEA Results Summary

  

Fig 1: Enrichment plot: KEGG\_INOSITOL\_PHOSPHATE\_METABOLISM      
 Profile of the Running ES Score & Positions of GeneSet Members on the Rank Ordered List

  

| SYMBOL | TITLE | RANK IN GENE LIST | RANK METRIC SCORE | RUNNING ES | CORE ENRICHMENT || 1 | INPP4B | na | 2804 | 0.306 | -0.0379 | No |
| 2 | SYNJ1 | na | 5250 | 0.215 | -0.0781 | No |
| 3 | IPMK | na | 7140 | 0.160 | -0.1101 | No |
| 4 | MINPP1 | na | 8104 | 0.136 | -0.1188 | No |
| 5 | PLCB3 | na | 9511 | 0.106 | -0.1445 | No |
| 6 | PLCB2 | na | 9762 | 0.100 | -0.1376 | No |
| 7 | PIK3CG | na | 9794 | 0.100 | -0.1245 | No |
| 8 | PIKFYVE | na | 9889 | 0.098 | -0.1134 | No |
| 9 | PTEN | na | 11228 | 0.075 | -0.1415 | No |
| 10 | PIK3CA | na | 11255 | 0.074 | -0.1318 | No |
| 11 | PIP4K2B | na | 11849 | 0.066 | -0.1397 | No |
| 12 | PLCD3 | na | 12804 | 0.052 | -0.1598 | No |
| 13 | PLCG1 | na | 12816 | 0.052 | -0.1528 | No |
| 14 | PLCG2 | na | 12987 | 0.050 | -0.1506 | No |
| 15 | PIK3C2B | na | 13343 | 0.046 | -0.1544 | No |
| 16 | INPP1 | na | 13409 | 0.045 | -0.1500 | No |
| 17 | INPP5J | na | 13978 | 0.038 | -0.1610 | No |
| 18 | ALDH6A1 | na | 14973 | 0.028 | -0.1857 | No |
| 19 | PLCD1 | na | 14981 | 0.028 | -0.1820 | No |
| 20 | INPP5K | na | 15229 | 0.026 | -0.1855 | No |
| 21 | IPPK | na | 15306 | 0.025 | -0.1842 | No |
| 22 | PIK3CB | na | 17199 | 0.007 | -0.2378 | No |
| 23 | PLCB4 | na | 17554 | 0.004 | -0.2475 | No |
| 24 | PIK3C2A | na | 19085 | -0.011 | -0.2900 | No |
| 25 | IMPA1 | na | 20128 | -0.024 | -0.3167 | No |
| 26 | PIK3CD | na | 20433 | -0.027 | -0.3217 | No |
| 27 | TPI1 | na | 20985 | -0.033 | -0.3329 | No |
| 28 | PIK3C3 | na | 21093 | -0.035 | -0.3311 | No |
| 29 | PIP5K1C | na | 21353 | -0.038 | -0.3333 | No |
| 30 | CDIPT | na | 22359 | -0.050 | -0.3552 | No |
| 31 | INPPL1 | na | 22619 | -0.054 | -0.3551 | No |
| 32 | INPP5E | na | 22632 | -0.054 | -0.3478 | No |
| 33 | PLCZ1 | na | 22984 | -0.059 | -0.3496 | No |
| 34 | INPP5A | na | 23168 | -0.062 | -0.3462 | No |
| 35 | PLCD4 | na | 23844 | -0.073 | -0.3553 | No |
| 36 | INPP4A | na | 24691 | -0.088 | -0.3674 | Yes |
| 37 | SYNJ2 | na | 24836 | -0.090 | -0.3588 | Yes |
| 38 | PIP4K2A | na | 24991 | -0.093 | -0.3502 | Yes |
| 39 | OCRL | na | 25116 | -0.095 | -0.3404 | Yes |
| 40 | MIOX | na | 25177 | -0.096 | -0.3286 | Yes |
| 41 | PI4KA | na | 26009 | -0.113 | -0.3366 | Yes |
| 42 | INPP5B | na | 26155 | -0.116 | -0.3245 | Yes |
| 43 | PI4KB | na | 26460 | -0.122 | -0.3160 | Yes |
| 44 | PIP4K2C | na | 26803 | -0.130 | -0.3075 | Yes |
| 45 | ITPK1 | na | 27265 | -0.142 | -0.3009 | Yes |
| 46 | ITPKA | na | 27757 | -0.154 | -0.2934 | Yes |
| 47 | IMPA2 | na | 28697 | -0.181 | -0.2950 | Yes |
| 48 | ITPKB | na | 29315 | -0.200 | -0.2846 | Yes |
| 49 | PIK3C2G | na | 30244 | -0.232 | -0.2788 | Yes |
| 50 | PLCB1 | na | 30796 | -0.254 | -0.2589 | Yes |
| 51 | ISYNA1 | na | 33328 | -0.422 | -0.2725 | Yes |
| 52 | PIP5K1A | na | 33731 | -0.475 | -0.2172 | Yes |
| 53 | PIP5K1B | na | 33965 | -0.515 | -0.1514 | Yes |
| 54 | PLCE1 | na | 34721 | -1.233 | 0.0002 | Yes |
Table: GSEA details [plain text format]

  

Fig 2: KEGG\_INOSITOL\_PHOSPHATE\_METABOLISM      
 Blue-Pink O' Gram in the Space of the Analyzed GeneSet

  

Fig 3: KEGG\_INOSITOL\_PHOSPHATE\_METABOLISM: Random ES distribution      
 Gene set null distribution of ES for **KEGG\_INOSITOL\_PHOSPHATE\_METABOLISM**
